# Updated guidelines for predictive biomarker testing in advanced non-small-cell lung cancer: a National Consensus of the Spanish Society of Pathology and the Spanish Society of Medical Oncology

**DOI:** 10.1007/s12094-019-02218-4

**Published:** 2019-10-09

**Authors:** P. Garrido, E. Conde, J. de Castro, J. J. Gómez-Román, E. Felip, L. Pijuan, D. Isla, J. Sanz, L. Paz-Ares, F. López-Ríos

**Affiliations:** 1 Department of Medical Oncology, Hospital Universitario Ramón y Cajal, Universidad Alcalá, IRYCIS, CIBERONC, Ctra. de Colmenar Viejo, km. 9100, 28034 Madrid, Spain; 2 Department of Pathology-Laboratorio de Dianas Terapéuticas, Hospital Universitario HM Sanchinarro, CIBERONC, Madrid, Spain; 3 Department of Medical Oncology, Hospital Universitario La Paz, Madrid, Spain; 4 Department of Pathology, Hospital Universitario Marqués de Valdecilla, Universidad de Cantabria, IDIVAL, Santander, Spain; 5 Department of Medical Oncology, Hospital Universitari Vall d’Hebron, Barcelona, Spain; 6 Department of Pathology, Hospital del Mar, Barcelona, Spain; 7 Department of Medical Oncology, Hospital Clínico Universitario Lozano Blesa, Zaragoza, Spain; 8 Department of Pathology, Facultad de Medicina, Instituto de Investigación Sanitaria del Hospital Clínico San Carlos (IdISSC), Universidad Complutense de Madrid, Madrid, Spain; 9 Department of Medical Oncology, Hospital Universitario 12 de Octubre, Madrid, Spain

**Keywords:** *ALK*, Biomarkers, Non-small-cell lung cancer, *EGFR*, *BRAF*, PD-L1, *ROS1*

## Abstract

In 2011 the Spanish Society of Medical Oncology (SEOM) and the Spanish Society of Pathology (SEAP) started a joint project to establish guidelines on biomarker testing in patients with advanced non-small-cell lung cancer (NSCLC) based on current evidence. As this field is constantly evolving, these guidelines have been updated, previously in 2012 and 2015 and now in 2019. Current evidence suggests that the mandatory tests to conduct in all patients with advanced NSCLC are for *EGFR* and *BRAF* mutations, *ALK* and *ROS1* rearrangements and PD-L1 expression. The growing need to study other emerging biomarkers has promoted the routine use of massive sequencing (next-generation sequencing, NGS). The coordination of every professional involved and the prioritisation of the most suitable tests and technologies for each case remains a challenge.

## Introduction

Non-small-cell lung cancer (NSCLC) is the solid tumour with the widest variety of potential therapeutic targets. It represents both a significant therapeutic opportunity and a challenge in predictive biomarkers determination. This third consensus statement update guidelines published in 2012 and 2015 focused on predictive biomarker testing in patients with advanced NSCLC [1, 2]. The current document is, supported by the Spanish Society of Pathology (SEAP) and the Spanish Society of Medical Oncology (SEOM).

## Requirements for testing an optimal biological specimen

Obtaining enough and optimal quality specimen for biomarkers in a particular patient should be a responsibility shared by the entire tumour board. In order to do this, it is important that the professionals involved have sufficient knowledge of the advantages and disadvantages of each technology. It would be very helpful to establish automated and routine channels that could provide a solution when one or all tests fail, always taking into account adequate response times. When conducting molecular and immunohistochemical (IHC) tests, it is important to consider the tumour percentage and the amount of tumour cells in the specimen, and also the pre-analytical variables [3]. Most of the samples obtained are small biopsy and/or cytology-type specimens (for example cell blocks, smears and liquid-based cytology). All of these sample types are suitable for IHC and molecular studies. The use of one or another will depend on the experience and capacity of each laboratory [4]. The first step for obtaining an adequate specimen is the time between sample removal out of patient and its early fixation. This is why having seamless communication between the specialists involved is essential, as well as the availability of optimised diagnostic techniques. The general requirements for a specimen to be optimal are conservation in 10% buffered formalin for 6–12 h for small biopsies and 24–48 h for surgical resections [5], and the presence of at least 50–100 viable cells for IHC studies or fluorescence in situ hybridisation (FISH). For real-time polymerase chain reaction (PCR) tests, a minimum 5% of tumour cells in NSCLCs are recommended [1, 6]. This percentage should be increased to 20–30% for direct next-generation sequencing (NGS) studies [7]. Direct smears that are air-dried or ethanol-based fixation and liquid-based cytology are also suitable for FISH and molecular testing, but it is compulsory to perform appropriate validation studies in each laboratory following previously described recommendations [8–10]. The use of cytology specimens has not yet been validated to determine the expression of programmed death ligand-1 (PD-L1), despite the good correlation observed between cytology smears and cell blocks with biopsies [11]. Tissue-sparing protocols are recommended [12, 13]. For liquid biopsies, the two key technical factors to maintain optimal preservation of circulating cell-free DNA (cfDNA) are the storage and shipping conditions of the sample, and the elapsed time between specimen extraction and processing [14].

## Which biomarkers should be tested in NSCLC and in which patients?

Table 1 summarises the essential biomarkers to be performed on tissue- and/or cytology-type samples from advanced NSCLC patients, including the predictive alterations and their testing methods.

**Table 1:** Essential biomarkers in NSCLC patients

Gene/protein	Predictive alteration	Methodology (in tissue)
*EGFR*	Mutation	PCR: sanger, real-time PCR and NGS
*ALK*	Rearrangement	IHC, FISH and NGS
*ROS1*	Rearrangement	IHC (screening), FISH and NGS
*BRAF V600*	Mutation	PCR: sanger, real-time PCR and NGS
PD-L1	Overexpression	IHC

*EGFR* epidermal growth factor receptor, *FISH* fluorescence in situ hybridisation, *H&E* haematoxylin/eosin, *IHC* immunohistochemistry, *NGS* next-generation sequencing, *NSCLC* non-small-cell lung cancer, *PCR* polymerase chain reaction, *PD-L1* programmed death ligand-1

### EGFR

**Fig. 1:**
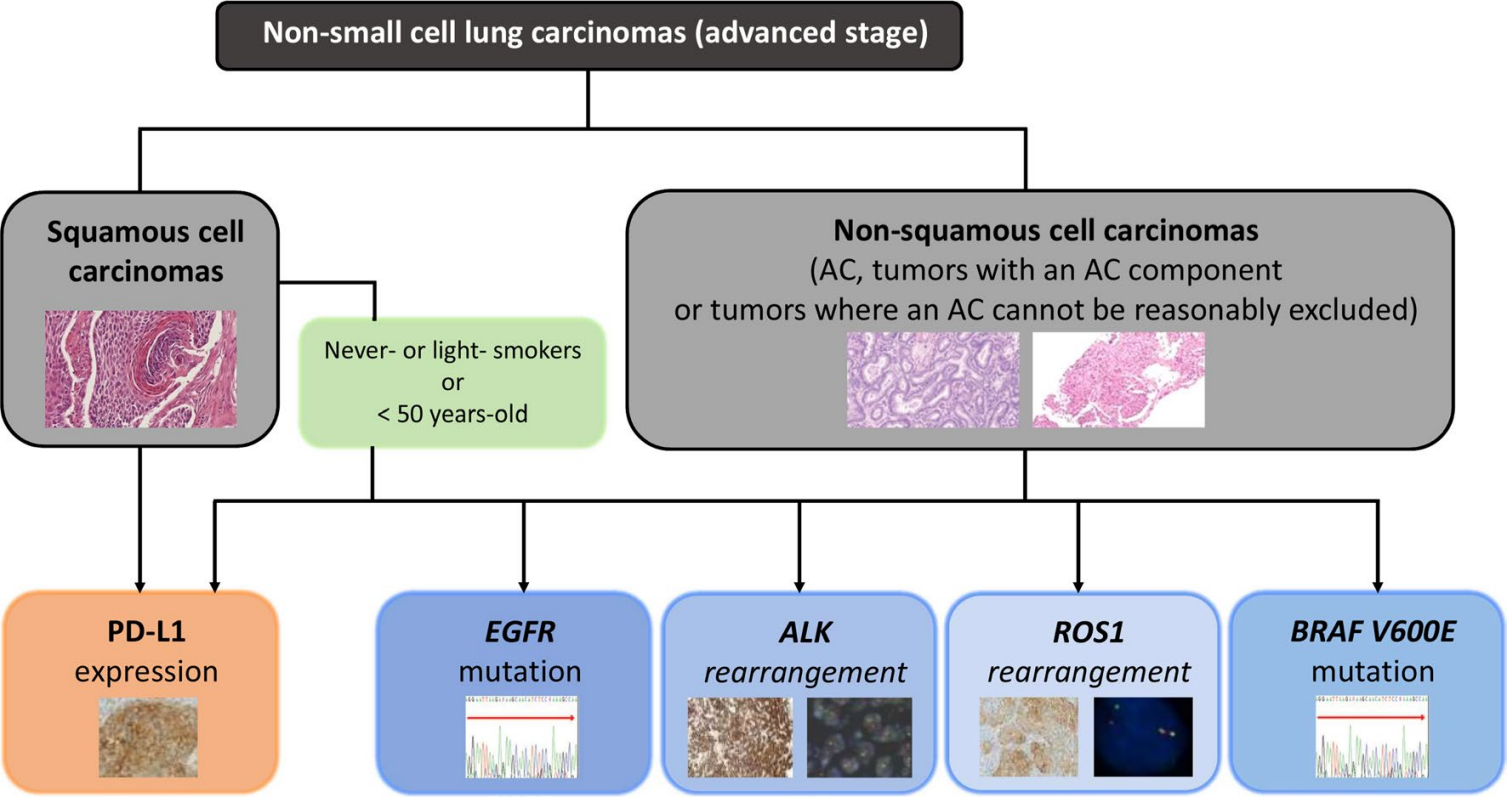
Diagnostic algorithm for biomarker testing in patients with advanced NSCLC. *AC* adenocarcinoma, *EGFR* epidermal growth factor receptor, *PD-L1* programmed death ligand-1
- The latest international consensus statements recommend that *EGFR* mutation tests should also be conducted on any small sample in which the tumour is poorly represented and in cases with an uncertain histological subtype;
- Lastly, an upfront liquid biopsy is not recommended if tissue is available. This procedure could be selected for determining the T790M mutation at disease progression.

In Spain, epidermal growth factor receptor (*EGFR)* mutations are present in 8–11% of advanced NSCLCs, and in 16–18% of lung adenocarcinomas [15]. The most common mutations (85–90%) are tyrosine-kinase inhibitors (TKIs) sensitivity mutations such as deletions in exon 19 and point mutations in exon 21. Other uncommon mutations may be clinically relevant (i.e. exon 20 insertions are typically intrinsically resistant to EGFR-TKI inhibitors and exon 18 alterations may be more sensitive to a specific TKI) [16]. EGFR-TKI inhibitor drugs are currently available, and administration as first-line therapy is standard in the main clinical guidelines [17], since these improve progression-free survival (PFS) and quality of life when compared to the administration of platinum doublet chemotherapy [17]. Therefore, the recommendations from the last SEOM/SEAP consensus statements are still valid [1]:

- *EGFR* mutation tests in patients with advanced NSCLC should be conducted for all adenocarcinomas, non-squamous non-small-cell histologies and squamous cell carcinomas in patients younger than 50 years of age and/or with no or low tobacco use (i.e. < 15 pack-years) (Fig. 1);

Most patients with an *EGFR* mutation who receive first- or second-generation EGFR-TKIs will progress, and the most frequent molecular mechanism for acquired resistance is *EGFR* T790M mutation, that occurs in 50–60% of cases [18]. In patients who present with an *EGFR* T790M mutation after progression on first-line treatment with a first- or second-generation EGFR-TKI, osimertinib has shown a higher PFS than a platinum/pemetrexed regimen (10.1 months vs. 4.4 months, respectively; HR 0.30) [19]. Based on this data, osimertinib is considered the treatment of choice for these patients. Resistance mechanisms are less well known when osimertinib is used as first-line treatment [20, 21]. Determination of *EGFR* T790M in tumour tissue and in cfDNA are both valid alternatives. If *EGFR* T790M testing in plasma is negative, a new biopsy is recommended whenever possible.

Recommendations:

- All individual *EGFR* mutations with a frequency higher than 1% should be tested in tissue and/or cytology-type samples;
- The pathologist should examine all available specimens and use the one with better cellularity and tumour proportion (biopsy or cytology) from the primary tumour or the metastases;
- High-sensitivity detection methods should be used, especially for *EGFR* T790M mutation testing (5% detection limit) [22]. The most recent recommendations from the American Society of Clinical Oncology/College of American Pathologists (ASCO/CAP) and the National Institute for Health and Care Excellence (NICE) suggest having two alternative methods to carry out a redundant molecular test, if necessary;
- When the objective is to select patients to receive a therapy, IHC techniques (including *EGFR* mutation-specific antibodies) or copy number analysis should not be used;
- If sufficient expertise is available, and if the extended biomarker panel is to be tested, it is preferable to determine the *EGFR* mutation with targeted NGS panels;
- Cell blocks and other cytological preparations tested in laboratories with experience are also suitable specimens.

### ALK

Anaplastic lymphoma kinase (*ALK*) rearrangements are present in 2–5% of advanced NSCLCs [17]. Due to the clinical benefit provided by targeted therapies in this disease, it is key of identify all patients with this molecularly driven type of lung cancer [23].

Recommendations:

- The histological types eligible for *ALK* rearrangement tests include all adenocarcinomas, carcinomas with non-squamous histological evidence and squamous tumours in patients younger than 50 years of age and/or with low or no tobacco use (i.e. < 15 pack-years) (Fig. 1) [24]. In some neuroendocrine carcinomas, ALK expression is intense but rearrangement cannot be detected in the sequencing test [25, 26]. The key methods for detecting *ALK* gene rearrangement are IHC, FISH, PCR and NGS [22, 27]. Actually, IHC is an equivalent alternative to FISH. In this regard [17, 24] IHC is a quick and cost-effective method to determine low prevalence biomarkers. Cell integrity can be assessed, and the method can be applied to different biological specimens, such as biopsies or cytological samples. Its use in cytology smears is quite controversial, although recent studies have proven the suitability of the method [28]. The most commonly used antibodies are D5F3 (Ventana *ALK* [D5F3] CDx Assay, Tucson, Arizona, USA) and 5A4 (Novocastra, Leica Biosystems, Buffalo Grove, Illinois, USA), although the latter is not included in a diagnostic kit [29]. The role of FISH as the optimal standard methodology is currently under discussion. The technical rationale is being assessed, as well as its interpretation of complex molecular mechanisms [30], although there are automated reader algorithms approved by the food and drug administration (FDA) that greatly increase reliability [29]. When there is a positive IHC as manifested by strong granular cytoplasmic staining with either of the 5A4 or D5F3 antibodies, the current recommendation is that confirmation by a second technique is not mandatory [22]. However, it is advisable to do so in cases that are inconclusive. This diagnostic redundancy is also helpful if unusual FISH patterns are found [31]. The methods based on NGS and RNA-assays are highly specific and there are numerous studies that demonstrate their value for detecting fusions in patients who show negative results with other techniques [32, 33].

Lastly, variant testing for specific rearrangements in *ALK*, which has been reported as a crucial element in the clinical response to specific inhibitors, does not yet have sufficient data for recommendation, although it could be useful in the future [34].

### ROS1

The c-ros oncogene 1 (*ROS1*) gene encodes a receptor with tyrosine kinase activity that appears to be translocated in approximately 1% of NSCLCs, especially in young, non-smoking patients. It is associated with adenocarcinoma histology, with the presence of a solid component and signet-ring cells. This histological profile is also typical of tumours harbouring an *ALK* translocation. In fact, both receptors have a 77% similarity in their ATP-binding domain.

Crizotinib is approved as a first- or second-line monotherapy in stage IV lung cancer patients with *ROS1* rearrangement [35–37]. Other drugs, such as ceritinib, brigatinib, lorlatinib and entrectinib, are being studied but they are not approved for this indication yet.

Recommendations:

- It is currently recommended to carry out *ROS1* testing in patients with advanced stage (IIIB-IV) lung adenocarcinoma, regardless of its clinical characteristics [17] (Fig. 1). *ROS1* testing is not recommended in squamous cell carcinoma, except in the context of patients with no or low tobacco exposure [17, 22].Essentially, there are three methodological approaches to detecting *ROS1* rearrangements: (a) IHC; (b) cytogenetic techniques, particularly FISH; and (c) molecular techniques, such as reverse transcription PCR (RT-PCR) or NGS [30, 38]. To determine *ROS1* translocation in clinical specimens, international guidelines recommend IHC as the screening method and confirmation of positive cases with another orthogonal method (cytogenetic or molecular) [22]. Currently there is no FDA-approved IHC assay for clinical routine, but there are two commercially available antibodies (D4D6, Cell Signalling Technology and SP384, Ventana Medical Systems) which show high sensitivity in most studies when compared to other techniques, in particular FISH or RT-PCR [22, 39]. However, according to the method and the criteria for positivity used, the specificity ranges from 70 to 100% [22, 39]. At present, there is no universally accepted system for how to score IHC results but it is recommended that the specimen includes at least 20 tumour cells and that each laboratory validates its own interpretation range [22, 24, 38, 40]. Moreover, it is important to consider that ROS1 expression without underlying rearrangement (false positives) has been described in nearly a third of tumours [41, 42]. The presence of other molecular abnormalities, such as *EGFR*, *KRAS*, *BRAF* or *HER2* mutations and *ALK* rearrangements, has also been identified in some of these tumours [43].
- Regarding FISH, usually considered as the gold-standard technique, the use of a dual-colour break-apart probes and a count of at least 50 tumour cells is recommended [22, 38–40]. A tumour should be considered positive when at least 50% of tumour cells have break-apart signals (separated by ≥ 1 signal diameter), and/or 3’ isolated signals (frequently marked with green fluorochrome) [38, 39]. False positives and false negatives have been described, attributable to both methodological and biological causes [38, 40]. In respect of this latter aspect, it is important to note that some commercial probes could not detect rearrangements due to their design, as is the case for the variant *GOPC*-*ROS1* [38, 44].
- Regarding RT-PCR and NGS (DNA or RNA-based), most published studies show high sensitivity and specificity data [33, 44, 45].

### BRAF

*BRAF* mutations can be found in approximately 2% of lung carcinomas, both in smokers and non-smokers. Most of these are adenocarcinomas and tumours with papillary growth (Fig. 1) [46, 47]. Nearly all studies find a 50% frequency for *BRAF* V600E mutations [48], although in a European study the frequency is as high as 83% [49]. Additionally, *BRAF* V600E mutations are mostly mutually exclusive with most druggable abnormalities present in this tumour [46, 50]. It should be noted that certain *BRAF* mutations can co-exist with *KRAS* mutations [50]. Following robust results from clinical studies with *BRAF* inhibitors, whether or not associated with *MEK* inhibitors, both the EMA and FDA have approved dabrafenib and trametinib treatment for patients with the V600E mutation [51].

Recommendations:

- It is currently recommended to study the *BRAF V600* mutation in all patients with advanced non-squamous NSCLC (Fig. 1) [17, 22]
- The *BRAF* test can be conducted with any PCR method, including NGS, but the methodology should always analyse exons 11 and 15 [46]. Along this lines, the FDA has included the panel Oncomine Dx Target Test^®^ (ThermoFisher, Mass, USA) in its approval [52].

### PD-L1

In randomised studies, immunotherapy with PD-1/PD-L1 (nivolumab, pembrolizumab, atezolizumab and durvalumab) and CTLA4 inhibitors (ipilimumab in combination with nivolumab) is shown to be effective in patients with advanced NSCLC [17]. PD-L1 is a type-1 transmembrane protein (B7-H1) that belongs to the B7 ligand family, which can be expressed both by haematopoietic cells (lymphocytes) and non-haematopoietic cells (tumour cells) [53]. In advanced NSCLC, overexpression of PD-L1 is predictive of clinical benefit with PD-1/PD-L1 inhibitor drugs. In metastatic disease, and as a first-line palliative therapy, it is clearly predictive of efficacy for monotherapy with pembrolizumab when PD-L1 ≥ 50% [54, 55]. In some studies, it is also predictive of efficacy for the combination of PD-1/PD-L1 inhibitors with chemotherapy [56, 57]. In pre-treated patients with advanced NSCLC, overexpression of PD-L1 is also predictive of efficacy with nivolumab, pembrolizumab and atezolizumab [17]. In general, there is a correlation between positive testing for the biomarker and efficacy, although this is a marker with a suboptimal negative predictive value [58].

The standard treatment of unresectable stage III NSCLC changed due to the positive results in terms of PFS and OS of the PACIFIC study [59]. This phase III double-blind, placebo-controlled trial randomized PD-L1 unselected patients with stage III, locally advanced, unresectable NSCLC who did not progressed after chemoradiotherapy in a 2:1 ratio to receive durvalumab or placebo every to 2 weeks for up to 12 months. PACIFIC allowed any level of PD-L1 expression and tumour tissue collection was not required. Nevertheless, the European Medicines Agency restricted the approval of durvalumab to treat patients with PD-L1 ≥ 1% tumour cell expression based on a post hoc exploratory analysis. Due to this, the determination of PDL1 status is now mandatory in unresectable stage III patients suitable to receive durvalumab once completed concurrent chemoradiotherapy in the absence of progressive disease.

Recommendations:

- PD-L1 expression by IHC is currently accepted as the only validated biomarker for anti-PD-1/PD-L1 therapy in unresectable locally advanced (based on a controversial EMA decision) and advanced NSCLC [60]. Thus, in clinical practice, it should always be part of the diagnosis algorithm in order to select the best treatment option.
- Evidence for the presence of the PD-L1 protein can be obtained in formalin-fixed paraffin-embedded tissue specimens. Regarding preanalytical conditions, the most critical step is an enough time of fixation (i.e. at least 6 h), but storage time could also be relevant (i.e. archival material fewer than 3 years is recommended) [61].
- PD-L1 is expressed at the membrane level, while intracytoplasmic expression is less frequent (not considered a positive result) and it is observed in tumour and/or immune cells.
- There are several PD-L1 clones available for IHC testing. The four most widely used in pathology labs are 22C3 and 28–8 by Agilent/Dako, which share the Autostainer LINK 48 diagnostic platform by Dako, SP263 by MedImmune/Ventana and SP142 by Spring/Bioscience/Ventana, which share the Ventana BenchMark diagnostic platform. For routine diagnostics, the most frequently used clones are any of the first three, since these have shown good expression correlation between them in several studies. With respect to the other clones, SP142 stains a lower proportion of tumour cells [62].
- With these four clones, a positive result for PD-L1 is evaluated according to the percentage expression in tumour cells (partial or full membrane expression) at any intensity. With SP142, the proportion of the tumoral area occupied by immune cells is also evaluated [61].
- In small biopsies, at least 50–100 viable cells should be tested in order to validate the test result.
- At present, this can also be conducted with cytology [11, 63], but there is no study available to date that establishes a relationship with treatment response despite the good correlation observed between direct smears or cell blocks with biopsies [11].
- Since indications change rapidly, it seems reasonable to recommend including all the quantifiable information (percentage of positive tumour cells and percentage of positive immune cells) in every report, and not only the qualitative value (positive vs. negative).

## Which other biomarkers in NSCLC are currently of interest?

Table 2 summarises other biomarkers to be performed on tissue- and/or cytology-type samples from advanced NSCLC patients, including its predictive alteration and the method for testing.

**Table 2:** Other biomarkers of interest in NSCLC patients

Gene	Predictive alteration	Methodology (in tissue)
*HER2*	Mutation	PCR: sanger, real-time PCR and NGS
	Amplification	FISH, NGS, real-time PCR
*MET*	Mutation	NGS
	Amplification	FISH, NGS, real-time PCR
*RET*	Rearrangement	FISH and NGS
*NTRK*	Rearrangement	IHC (screening) and NGS
TMB	Mutations*	NGS

*FISH* fluorescence in situ hybridisation, *IHC* immunohistochemistry, *NGS* next-generation sequencing, *NSCLC* non-small-cell lung cancer, *PCR* polymerase chain reaction, *TMB* tumour mutation burden

*Measurement of somatic mutations present in tumour cells

### HER2

The presence of *HER2* abnormalities in advanced NSCLC patients can also be ancillary to targeted therapy, but the data to date is controversial, both from the clinical viewpoint and from the biomarker perspective. Two main deregulation mechanisms have been described that are mutually exclusive with other oncogenic abnormalities: (a) mutations of which 90% are in the kinase domain (exon 20), the most frequent being p.A775_G776insYVMA insertion, especially in adenocarcinomas, with an approximate frequency of 3% [64]; and (b) amplification/overexpression that occurs in a similar percentage of, and can overlap with mutations in 11% of cases [65]. *HER2* mutations seem to be the best clinical benefit predictors [65]. It also should be noted that squamous cell lung carcinomas can present *HER2* mutations, but outside the kinase domain, with certain clinical benefit data when treating with afatinib [66]. As a summary, the following points can be useful:

- As an isolated biomarker, HER2 IHC may not be sufficient to select patients who can benefit from anti-HER2 therapies [67, 68];
- *HER2* mutations identified by NGS could give access to investigational targeted drugs in clinical trials [69];
- *HER2* amplification has been described as a resistance mechanism after therapy with EGFR-TKIs and also as a “de novo” alteration in pan-negative adenocarcinomas [16, 70].

### MET

The *MET* gene encodes a tyrosine kinase receptor activated by its specific natural ligand: the hepatocyte growth factor receptor (HGFR). *MET* amplification (3–7%), as well as overexpression (25–75%), implies a worse prognosis for the patient, with the cut-off point with predictive value in dispute. Ten to twenty percent of patients with *EGFR*-mutated tumours acquire EGFR-TKI resistance through *MET* amplification, and the therapeutic implications of this are being explored [16]. Moreover, *MET* exon 14 (*MET*ex14) mutations are identified in approximately 3% of NSCLC cases. These are frequently concomitant with gene amplification, and present specific clinicopathological features (e.g. elderly patients, sarcomatoid histology or adenocarcinoma) [71, 72]. These mutations are predictive of benefit with specific MET-TKIs (crizotinib, tepotinib or capmatinib) [72, 73]. The preferred technique should be NGS. Sanger sequencing can detect *MET*ex14, but large deletions or low allelic frequency can hinder sensitivity. Quantitative RT-PCR (qRT-PCR), a method based on messenger RNA (mRNA), is sensitive and specific; therefore, it can be appropriate for selecting *MET*ex14 as a single gene test.

### RET

Two main activation mechanisms have been described for the oncogenic kinase *RET*: point mutations and genetic rearrangements. Activating point mutations are most common in medullary thyroid cancer. *RET* fusions are observed in 10% of papillary thyroid cancers, 1–2% of NSCLC cases and other cancer subtypes, including colorectal, pancreatic and breast cancers [74]. In NSCLC, *RET* fusion presents mainly in adenocarcinomas of non-smoker patients, and the partner that is most frequently associated in this setting is *KIF5B.* In lung adenocarcinoma, the presence of calcifications in the form of psammoma bodies could be indicative of the possibility of finding this alteration [75]. Some multiple TKIs have shown activity in NSCLC with *RET* fusion, as well as in other cancer types. Recently, two molecules especially designed as strong and selective inhibitors, BLU-667 and LOXO 292, have shown promising activity in *RET*-positive NSCLCs, as well as in other tumours with *RET* mutations or rearrangements [74, 76]. NGS-based panels, including *RET*, may be more suitable than PCR-based diagnostic methods, as the former can detect abnormalities in multiple genes simultaneously. The FISH technique is also a valid alternative in this scenario [74, 77].

### NTRK

The tropomyosin receptor kinase family is encoded by three genes (*NTRK1*, *NTRK2* and *NTRK3*), and its activation by rearrangement is targetable. Several drugs are the subject of clinical trials, and at least two of them are approved or in the process of approval: larotrectinib (LOXO-101, a selective inhibitor) and entrectinib (also a ROS1 and ALK inhibitor) [78]. There is a very small proportion of lung carcinoma patients (especially with adenocarcinomas) that present rearrangements in *NTRK1*, *NTRK2* or *NTRK3* [79]. Although early studies showed higher percentages, recent publications suggest a prevalence of less than 1% [78]. It is worth stating that these three abnormalities are mutually exclusive and that they are not present together with the main targetable abnormalities in lung adenocarcinomas [70, 78]. Two strategies are recommended for detecting these abnormalities: (a) NGS with a panel that includes testing for the three genes and with mandatory RNA testing to avoid false negatives; (b) IHC screening, with subsequent confirmation of every positive result by FISH or NGS [80, 81]. The IHC assay should be used according to the recently released ESMO recommendations [81].

### TMB

The tumour mutation burden (TMB), also known as mutation load, is an independent biomarker for immunotherapy in many types of tumours including lung cancer [82, 83]. TMB refers to the number of somatic mutations present in the tumour, after eliminating polymorphisms and germline mutations from all variants expressed per megabase (MB) in the studied exome. The mutations acquired by tumour cells can be reflected as an abnormal protein structure, and consequently, in the expression of neoantigens that can be related to the immunotherapy response. With regard to testing, targeted NGS is considered to be a good alternative to more complex massive sequencing [84]. Although this biomarker is not yet validated for clinical practice, it may be helpful in selecting patients for immunotheraphy as NSCLCs with a high mutation burden are more sensitive to these treatments [83]. Furthermore, there are implementation difficulties due to the tissue requirements, the definition of TMB, the need for validating interconnectivity between different NGS studies, with the heterogeneity of the numbers of included genes, horizontal coverage, the required optimal depth, and the chemical sequencing type, etc. and also, and more importantly, because the algorithms are continuously developing [84]. If eventually drugs are approved based on TMB cut-offs, the harmonization efforts underway could be very useful [84].

### Other biomarkers

The *KRAS* gene appears to be mutated in about 20% of all cases of NSCLC, especially in adenocarcinomas and smokers. Although its prognostic value has not been clearly demonstrated, it is the most common oncogenic mutation in lung cancer. In fact, many treatment strategies (such as the farnesyl transferase inhibitors, MEK and CDK4/6) have failed in this context [85]. For this reason, *KRAS* testing is currently not indicated as an individual test but it is appropriate that the study of the *KRAS* gene is included in extended panels [21, 22].

With regards to other potential biomarkers predictive of an immune response, the microsatellite instability and the immune microenvironment study should be highlighted, from the viewpoint of RNA expression and tissue determination of multiple immune cells [17, 58, 86, 87].

## How to prioritise the use of biological specimens for an accurate diagnosis

Most previous recommendations regarding sample prioritisation and its preservation for multiple biomarkers testing in advanced NSCLC patients are still valid [1, 13]. However, there are several new aspects that require an update on sample-sparing procedures [22, 24, 40].

Regarding histological diagnosis, it is still advisable to use the smallest amount of tissue for tumour typing, with a reasonable use of classificatory IHC [88]. This means using no more than two markers (i.e. TTF1 and p40) in cases without any clear morphological differentiation. It is worth noting that a different degree of TTF1 positivity has been described for adenocarcinomas, depending on the clone used (for example, the 8G7G3/1 antibody shows higher specificity and lower sensitivity than other clones) [89].

Regarding the testing of both molecular and immune biomarkers (see previous sections), it is still important to remember two principles: (a) the fewer times paraffin-embedded material (tissue or cytological as cell blocks) is placed in a microtome, the more will be spared; and (b) the order of biomarker prioritisation is important, as the tissue can be depleted [13]. To meet at least the first principle, testing should be always planned in advance for every NSCLC patient.

**Fig. 2:**
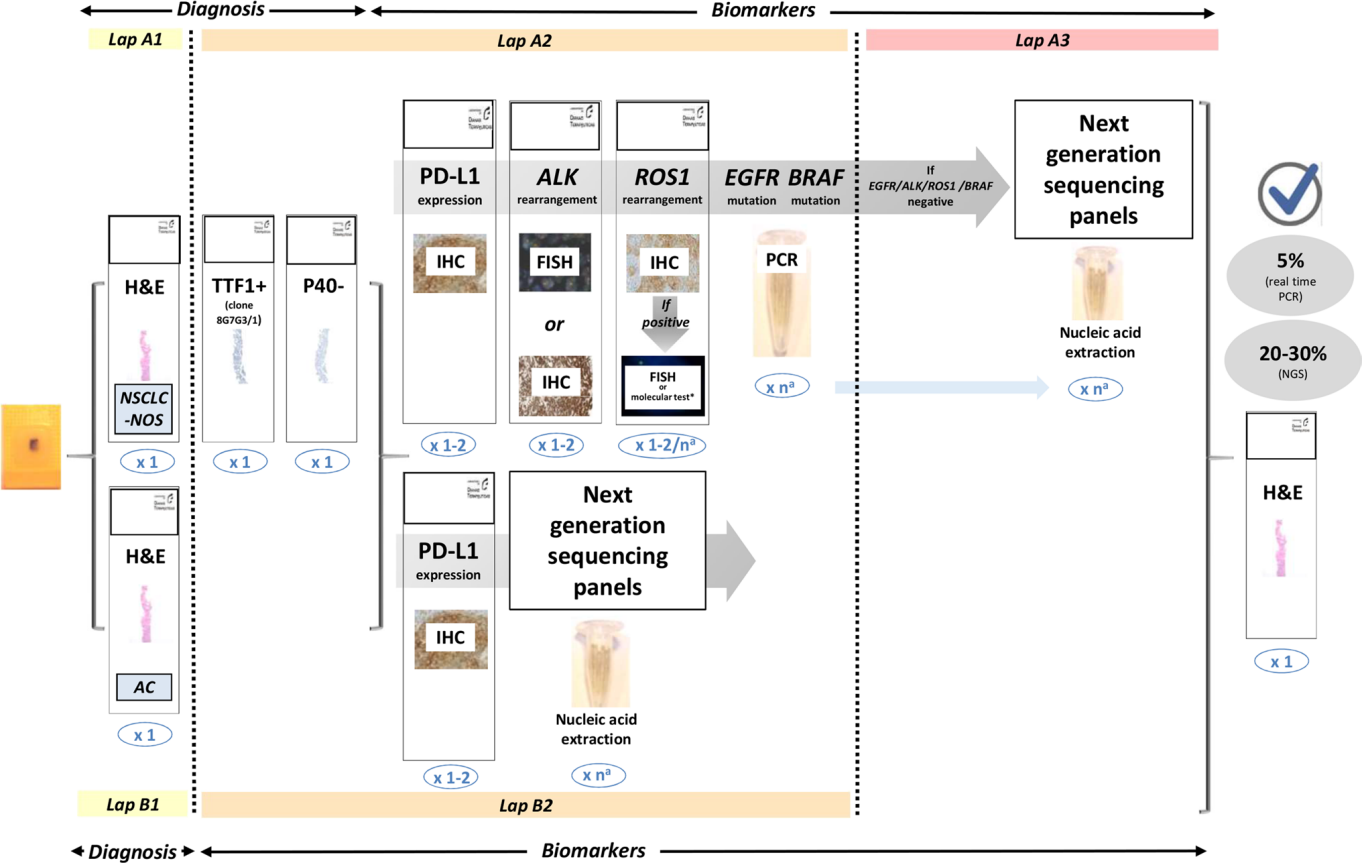
Protocol for multiple biomarker testing on samples from patients with advanced NSCLC. The number of sections for each test is shown in blue. ^a^The requirements for nucleic acid extraction for individual molecular testing or for extended genetic panels (NGS) are variable. *AC* adenocarcinoma, *EGFR* epidermal growth factor receptor, *FISH* fluorescence in situ hybridisation, *H&E* haematoxylin and eosin, *IHC* immunohistochemistry, *NGS* next-generation sequencing, *NSCLC-NOS* non-small-cell lung carcinoma – not otherwise specified, *PCR* polymerase chain reaction, *PD-L1* programmed death ligand-1 (Adapted protocol from international guidelines ASCO/CAP, ESMO and NCCN [17, 22, 24, 40]. Figure modified from Conde et al. (confidential, submitted))

Regarding the molecular biomarkers to be analysed, apart from *EGFR* mutations and *ALK* translocations, it is currently mandatory to include testing for *ROS1* rearrangements and *BRAF* mutations [17, 24]. The study of biomarkers such as *MET*, *RET*, *HER2, NTRK* and *KRAS* as individual tests is currently not indicated, but instead it is advised to include these biomarkers in extended panels performed either initially in all advanced NSCLCs or when previous *EGFR/ALK/ROS1/BRAF* testing is negative [17, 24]. The recommended protocol to follow is shown in Fig. 2, which includes the new biomarkers, detailing the recommended techniques and showing the two alternative pathways. Both routes are equally valid but upfront NGS could be a more cost/effective approach [90]. An aspect that undoubtedly improves overall quality is to use validated tests and to take part in quality control programs (see below).

One issue under discussion and without reference in international guidelines is how to incorporate immune biomarkers. The proposed sequence of previous steps includes one slice (or two) for PD-L1, together with the slides needed for *ALK* and *ROS1,* or before nucleic acids extraction [61].

Other issue being debated is whether the biomarkers should be reflex tested (by the pathologist at diagnosis) or in response to the clinical request. Ideally, this should be done as reflex testing so the pathologist can prioritise the sample, avoid the need to review preparations when the molecular tests are requested, and minimise response time, although this is not always possible [91, 92].

The whole concept of sample prioritization and multidisciplinary coordination should be organized through a molecular tumour board [93].

## The role of NGS in NSCLC

Following the discovery of new low-frequency abnormalities, there is an increased need for multigene testing, as opposed to single approaches. Testing should include *RET*, *HER2*, *NTRK*, *KRAS* and *MET* for cases in which the usual oncogenic drivers (*EGFR*, *ALK*, *ROS1* and *BRAF*) give a negative result and whenever an adequate technique is available [24]. The advantage of ultrasequencing and transcriptome analysis is the possibility of conducting mass screening without any loss of sensitivity and specificity, reducing the use of minimal biological specimens.

The development of this technique results in three categories for biomarker classification: (a) key biomarkers that should be tested to identify patients who are to be treated with approved therapies; (b) additional biomarkers that are desirable for identifying those patients who can benefit from clinical trials; and (c) other biomarkers that, at present, are only used in research and are not used in clinical practice (exome, genome, transcriptome) [22].

Biomarkers can be tested simultaneously with NGS. This technique is capable of detecting not only point mutations or insertions/deletions (indels) but also rearrangements and copy number variations, as well as a wide range of structural variants [30]. As mentioned previously, NGS appears to be a optimal technique for TMB testing [84].

The fact that most available specimens in routine healthcare are fixed in formalin and embedded in paraffin, and that double testing for DNA and RNA is necessary, is decisive when considering whether a technique is ideal for clinical use. Additionally, the specimens most commonly available for lung cancer have a low tumour cell content. In fact, most recommended techniques are those that are capable of detecting molecular abnormalities in samples with at least 20–30% of cancer cells [24].

There are studies that show very good concordance data between different technical solutions based on paraffin-embedded tissue. All these procedures urgently require quality control on the pre-test fixation parameters, as well as control of tumour cellularity and quality control for the nucleic acids [94]. Although there are advantages and limitations associated with amplicon-based and hybrid capture solutions, the most important thing to keep in mind is the need to use RNA when looking for druggable fusions to avoid a significant risk of false negatives [95].

## The role of liquid biopsy in NSCLC

Tumour biopsies are often insufficient for molecular study or are impossible to obtain. This is why liquid biopsies have been proposed as an alternative. It has many advantages, as it is a minimally invasive technique that can be used at diagnosis or during follow-up. [96, 97]. Circulating tumour cells (CTCs), circulating tumour DNA (ctDNA), circulating exosomes, platelet RNA, and circulating tumour RNA (ctRNA) are included in the definition of liquid biopsy. ctDNA represents the whole genomic picture of the tumour and is used in current clinical practice for liquid biopsies to test for genetic and epigenetic abnormalities specific to the tumour [14]. It can be detected in blood and also in urine, pleural fluids and saliva, among others [98].

Test methods for ctDNA can have a high specificity [14]. Therefore, when a mutation is detected in a clinical setting, it can be used to determine a targeted therapy. Since levels of ctDNA vary significantly and can be as low as 0.01% of all cfDNA, detection techniques must have a high sensitivity in order to detect the DNA from tumour cells, from 15 to 0.01% being the most widely used [98]. These techniques include ARMS (amplification refractory mutation system) PCR, qPCR, digital PCR (dPCR), BEAMing (beads, emulsions, amplification and magnetics) and the recommended NGS technique when *ALK* and *ROS1* fusions are to be tested.

From a clinical perspective, these methods offer alternative diagnostic techniques when a tissue biopsy is insufficient or not viable to determine *EGFR* T790M resistance mutation in NSCLC patients who harbour *EGFR* mutations, and also when the disease progresses. Nevertheless, a negative result with liquid biopsy requires testing with conventional techniques, such as tumour biopsy. Although validation and clinical usefulness are not sufficiently determined as yet, this is a promising technique for diagnosing other molecular abnormalities and their resistance mechanisms. It offers different possible applications, such as response monitoring, tumour recurrence detection, determination of residual disease after full tumour resection, early detection of lung cancer and for immuno-oncology [14].

## Main requirements for implementing optimal quality control

Quality test control is important, necessary and should be incorporated into the quality plan of the laboratory or service conducting the tests. In Spain, it is recommended that the lab has an ISO 9001 certification, and also that the different tests be accredited by the UNE-EN ISO15189 standard that has started to be enforced by pathology and molecular laboratories and evaluated by the Spanish National Certification Entity (ENAC).

The roadmap of processes and quality indices should include (a) the staff involved (technicians, biologists, pathologists etc.) and their training, experience and standard operating procedures; (b) instrumentation, with CE certifications for use and maintenance; and (c) reagents. For more information, the SEAP [99], CAP [22], and Association of Directors of Anatomic and Surgical Pathology (ADASP) recommendations can be reviewed [100].

As a summary outline, the controls for every test can be: (a) internal, such as positive and negative controls associated with each test; (b) external, such as quality control schemes (SEAP, EMQN, UK-NEQAS) (Table 3); and (c) results control, to verify that the percentage of mutations found corresponds to the frequency described depending on the type of tested samples. To facilitate this last control, it is advised to take part in case registration programs set up in collaboration with SEAP (Lungpath or ALKanza) in order to compare results with those obtained in similar hospitals.

**Table 3:** Examples of european quality assurance shemes

Supplier	Name	Starting material	Aim	Format
EMQN	Molecular testing of cfDNA in plasma for *EGFR* gene mutations (pilot)	Plasma containing cfDNA	Mutations in the *EGFR* gene	Five mock clinical cases with matching samples
	Molecular testing in lung cancer	Mix of real tissue and artificial FFPE materials	Mutations in the *EGFR*, *PIK3CA*, *KRAS* and *BRAF* genes	Ten mock clinical cases with matching samples
	DNA Sequencing–NGS (vSomatic)	DNA sample derived from FFPE material	Any NGS strategy can be used	One mock clinical case with matching samples
	Oncogene panel testing	Rolled sections of FFPE materials	Mutations in the *EGFR*, *PIK3CA*, *KRAS*, *HRAS*, *NRAS*, *KIT*, *TP53* and *BRAF* genes	Three mock clinical cases with matching samples
ESP	*ALK* FISH	Slides	*ALK* rearrangements	Five resections, five digital cases
	ALK IHC	Slides	*ALK* rearrangements	Five resections
	*EGFR*, *KRAS* (optional), *BRAF* (optional)	Slides/rolled sections	Mutations	Ten resection specimens, possible cell-line
	*ROS1* fish	Slides	*ROS1* rearrangements	Five resections or possibly cell-lines, five digital cases
	ROS1 IHC	Slides	*ROS1* rearrangements	Five resections or possibly cell-lines
	PD-L1	Slides	PD-L1 overexpression	Eight resections (TMAs) and four digital cases
	*MET* EQA scheme (ex 14 skipping) for DNA and RNA	Slides/rolled sections	*MET* exon 14 mutations	Five resections
NordiQC	Companion PD-L1	Slides	PD-L1 overexpression	One preparation with multiple cases and one in-house case
SEAP	ALKanza MODULE	Slides	*ALK* rearrangements	One slide with four cases + one in house
	*EGFR*	Slides/rolled sections	*EGFR* mutations	Four consecutive slides
UKNQEQAS	NSLCC ALK IHC	Slides	*ALK* and *ROS1* rearrangements	One slide with several cases + one in house
	NSLCC *ALK/ROS1* FISH (pilot)	Slides	*ALK* and *ROS1* rearrangements	One slide with several cases + one in house
	NSLCC PD-L1 IHC (pilot)	Slides	PD-L1 overexpression	One slide with several cases + one in house
Gen QA	Lung cancer	Slides/rolled sections	*EGFR, ALK* (optional), *KRAS* (optional), *BRAF* (optional)	5–4 cases
	Circulating tumour DNA (pilot)	Plasma	*EGFR* mutations	Five cases
	Additional lung cancer biomarkers	Slides/rolled sections	*ROS1*, *RET* and *MET* (amplification)	Four cases

*cfDNA* cell-free DNA, *EGFR* epidermal growth factor receptor, *FFPE* formalin-fixed paraffin-embedded, *FISH* fluorescence in situ hybridisation, *IHC* immunohistochemistry, *NGS* next-generation sequencing, *PD-L1* programmed death ligand-1, *TMA* tissue microarrays

The most frequent quality indices are: (a) response time, with around 7–10 working days recommended, on the understanding that this refers to having all biomarker results available within this time frame, both from individual tests and targeted NGS; (b) results from previously described quality controls; and (c) discrepancy/error analysis. Therefore, the creation of multidisciplinary committees for analysing the molecular diagnoses will facilitate the establishment of these indices. Table 4 specifies the information that should be contained in the results report for any biomarker test.

**Table 4:** Proposed pathology results report

Identification of the patient and the doctor who ordered the test (or, failing that, the authorised person)
Pathological diagnosis
Type of specimen submitted:
Previous treatment (yes/no) Time of biopsy (initial/relapse/progression) Date on which the specimen was collected
The external code in the case of referral centres
The medium in which the specimen was received (fresh, frozen, paraffin-embedded, etc.)
The anatomical origin of the specimen
The order date, the specimen receipt date and the date on which the results were issued
The biomarker test method used, specifying detectable mutations and/or other abnormalities. In the case of commercial kits, the commercial name, the batch number and whether they are an approved ‘in vitro diagnostics’ product should be stated
The quality of the sample, specifying the percentage of cancer cells and whether the sample was enriched by micro- or macrodissection, as well as DNA concentration and purity
Comments about the adequate or inadequate nature of the sample
The test result, defining the type of molecular abnormality detected or the absence of molecular abnormalities
Identification of the professional responsible for the test (all phases)
Identification of the laboratory supervisor (optional)
Any additional information or comments of interest to the doctor who ordered the test
Accreditation or participation in quality programs

## Conclusions

The mandatory tests for every patient with advanced NSCLC are *EGFR* and *BRAF* mutations, *ALK* and *ROS1* rearrangements [17, 24, 40], and PD-L1 expression [17, 40]. However, the growing need to study emerging biomarkers (*HER2*, *MET*, *RET, NTRK* and TMB) warrants the establishment of a routine and more comprehensive molecular assessment with targeted NGS. The coordination between all professionals and prioritisation of the proper tests and technologies for each case remains a challenge. Thus, adequate multidisciplinary communication is essential in order to provide the information within the required time frame, with the required quality and at a reasonable cost.
